# Supplementary material for: Altered histone modifications in Aedes aegypti following Rift Valley fever virus exposure
Source: bioRxiv. 2025 Sep 16:2025.09.11.675494. Preprint. [Version 2] doi: 10.1101/2025.09.11.675494 (PMC12458330; doi:10.1101/2025.09.11.675494)
Supplement: Supplement 1 — S1 Table. RNA-Seq raw reads with Q20/Q30 scores, insert sizes and alignment rates. S2 Table. RNA-Seq DESeq2 analysis for all samples and timepoints. S3 Table. Normalized Enrichment Score Table for RVFV v BF time course. S4 Table. CUT&RUN-Seq raw reads with Q20/Q30 scores, insert sizes and alignment rates. S5 Table. H3K27ac DiffBind output for genes within 2kB of TSS. S6 Table. H3K9me3 DiffBind output for genes within 2kB of TSS. S7 Table. RVFV v BF DEGs with proximal H3K27Ac peaks at the 7dpe S8 Table. CI/GLI promoter regions identified using HOMER. S1 Fig. MA plots of RNA-Seq differential expression data. S2 Fig. GO-GSEA of RVFV v BF RNA-Seq differential expression data. S3 Fig. Over-representation analysis of all Ae. aegypti genes with Ci/Gli motif. S4 Fig. GO-GSEA of BF vs SF RNA-Seq transcript differential expression data. S5 Fig. H3K27ac and H3K9me3 peak profiles. Top row: H3K27ac Input-subtracted peak heatmaps of aligned reads at 1, 3 and 7 days post-treatment show global trends within 2 kB of TSS (x axis). Y-axis indicates RPGC (reads per genome coverage) of input-subtracted read alignments. Notice the different Y-axis scales. Bottom row: H3K9me3 Input-subtracted peak heatmaps. S6 Fig. RVFV vs BF datasets: GSEA of GOIs proximal to H3K27Ac and H3K9me3 marks. S7 Fig. GO categories for genes proximal to H3K27ac marks change over time. A. GO terms for genes within 2kB of TSS (MACS2 peak calls) displayed using over-representation analysis. X axis shows collection day (d1, d3, or d7), BF, bloodfed, SF, sugar-fed. Number in parentheses below the sample name indicates the number of macs2 peaks considered in the analysis. This is a qualitative analysis and does not indicate statistically significant differences between groups. B. Transition of GO terms over time in BF midguts. GO terms for genes within 2kB of TSS (DiffBind peak calls) displayed using over-representation analysis. S8 Fig. BFvSF 1 dpf: Relationship of H3K27ac peaks and DEGs. Coordinates for DEG start si [file media-1.zip › CutRun_ms_Suppl_Figs20250808.pptx]

## Slide 1
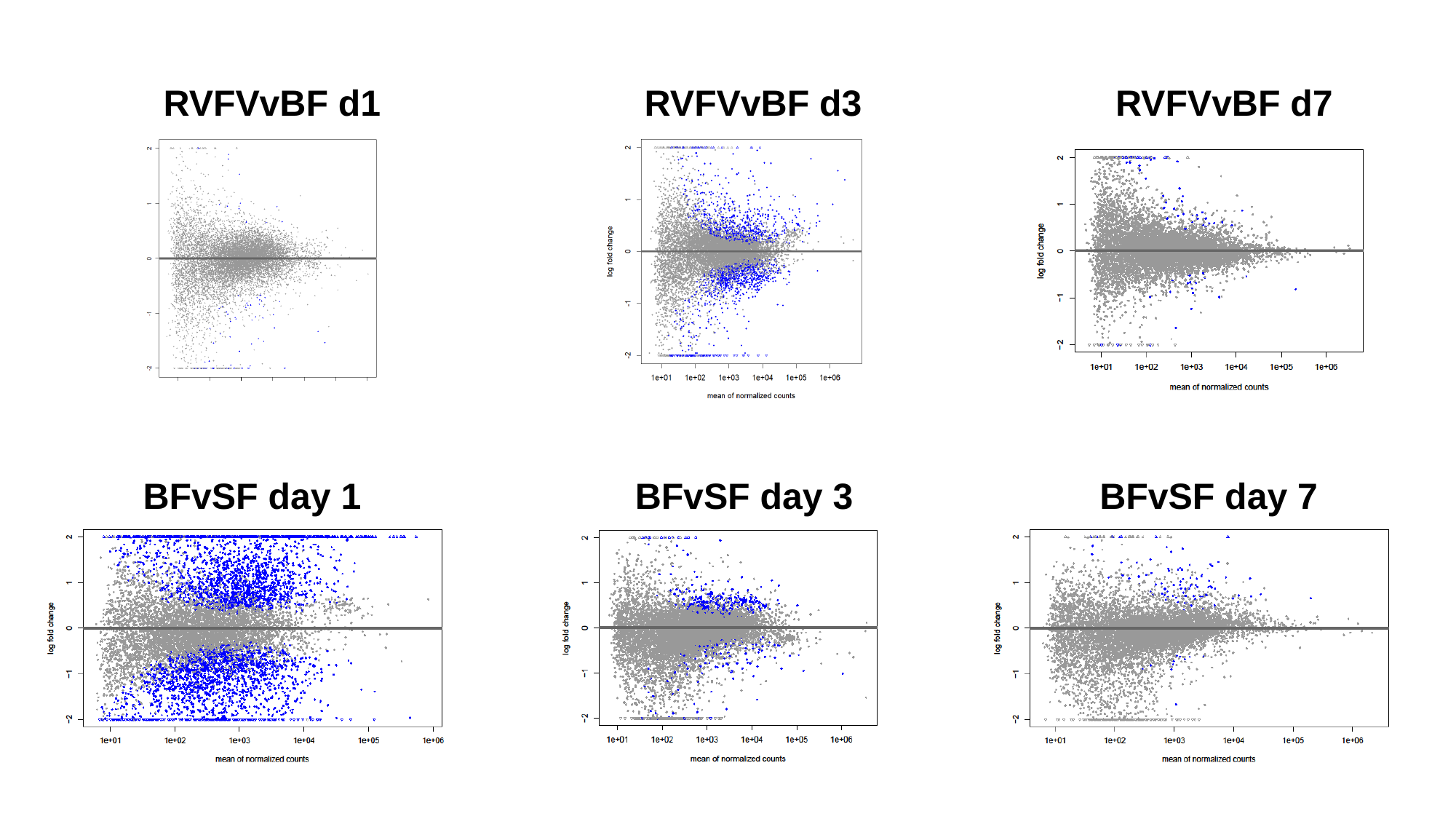

RVFVvBF d1 RVFVvBF d3 RVFVvBF d7
 BFvSF day 1 BFvSF day 3	 BFvSF day 7

## Slide 2
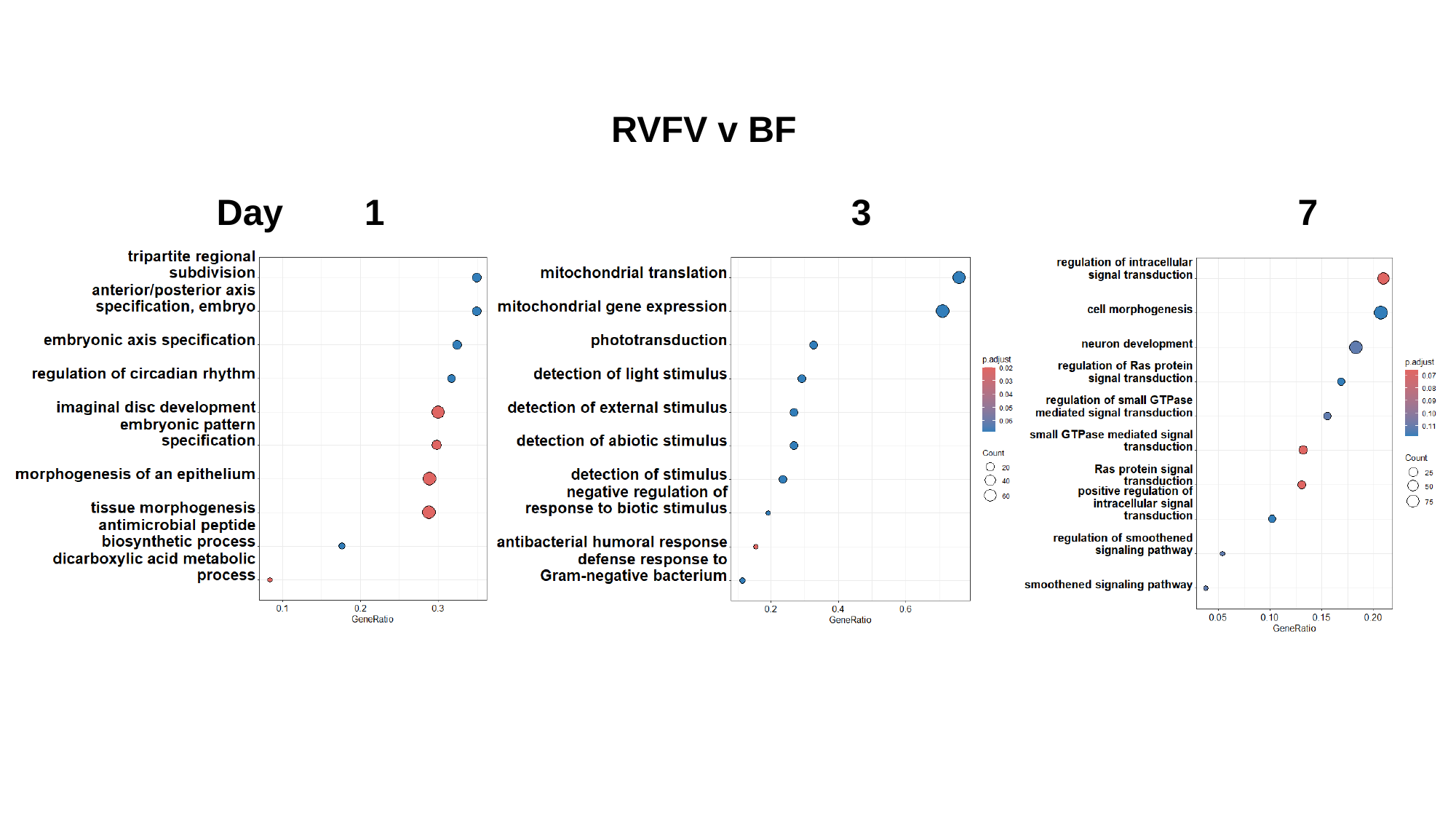

RVFV v BF
Day 1 3 7

## Slide 3
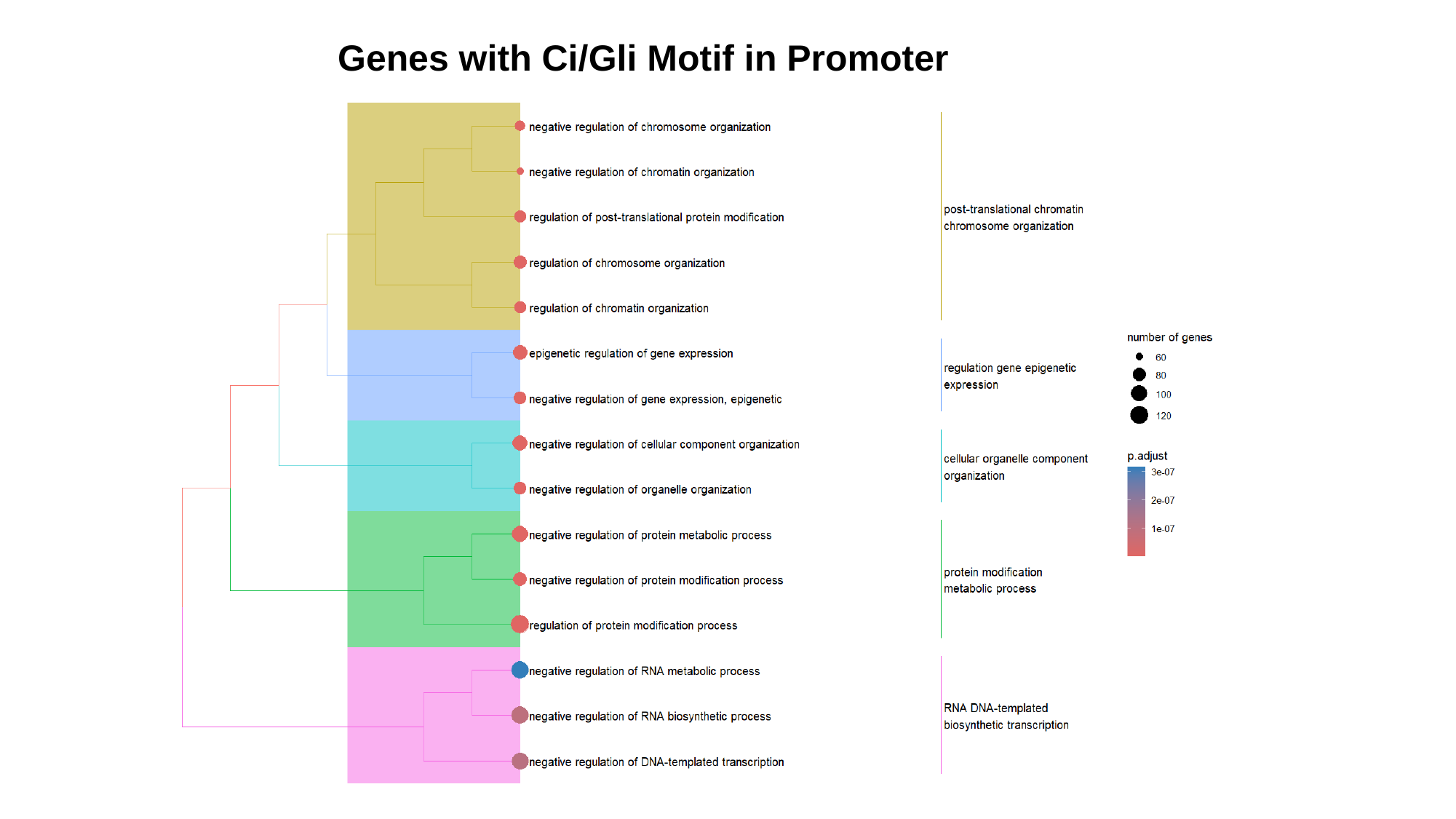

Genes with Ci/Gli Motif in Promoter

## Slide 4
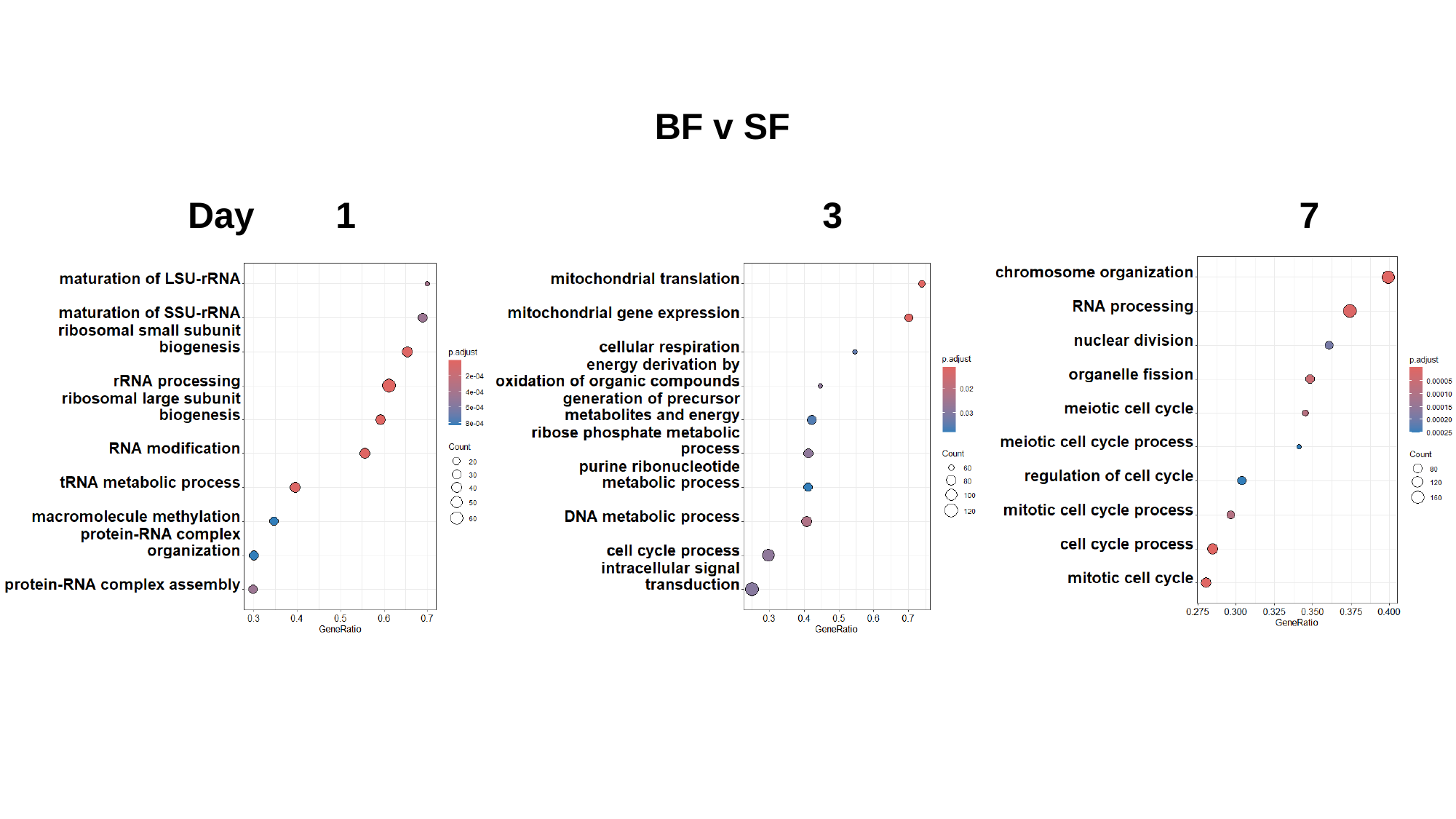

BF v SF
Day 1 3 7

## Slide 5
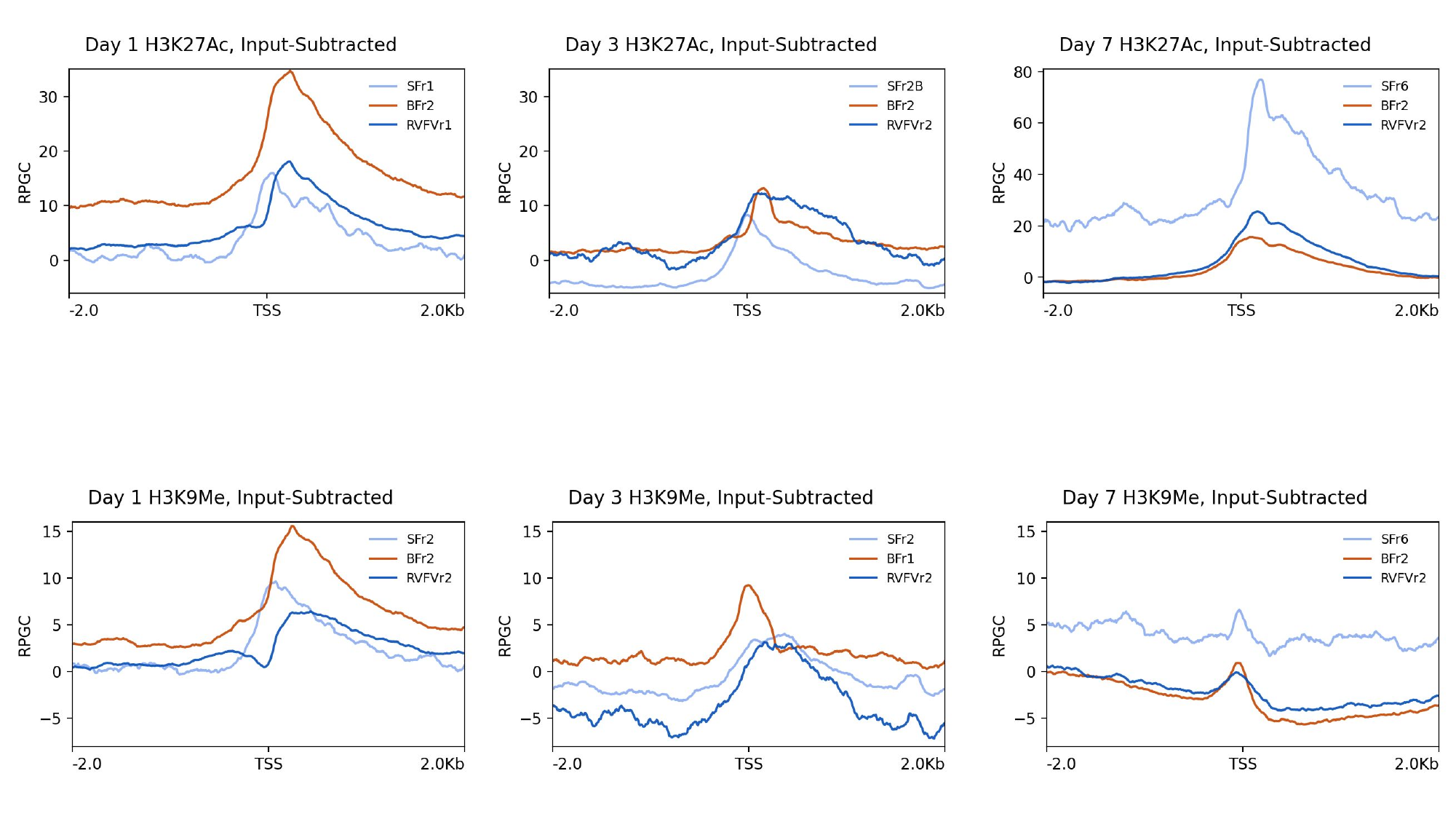

## Slide 6
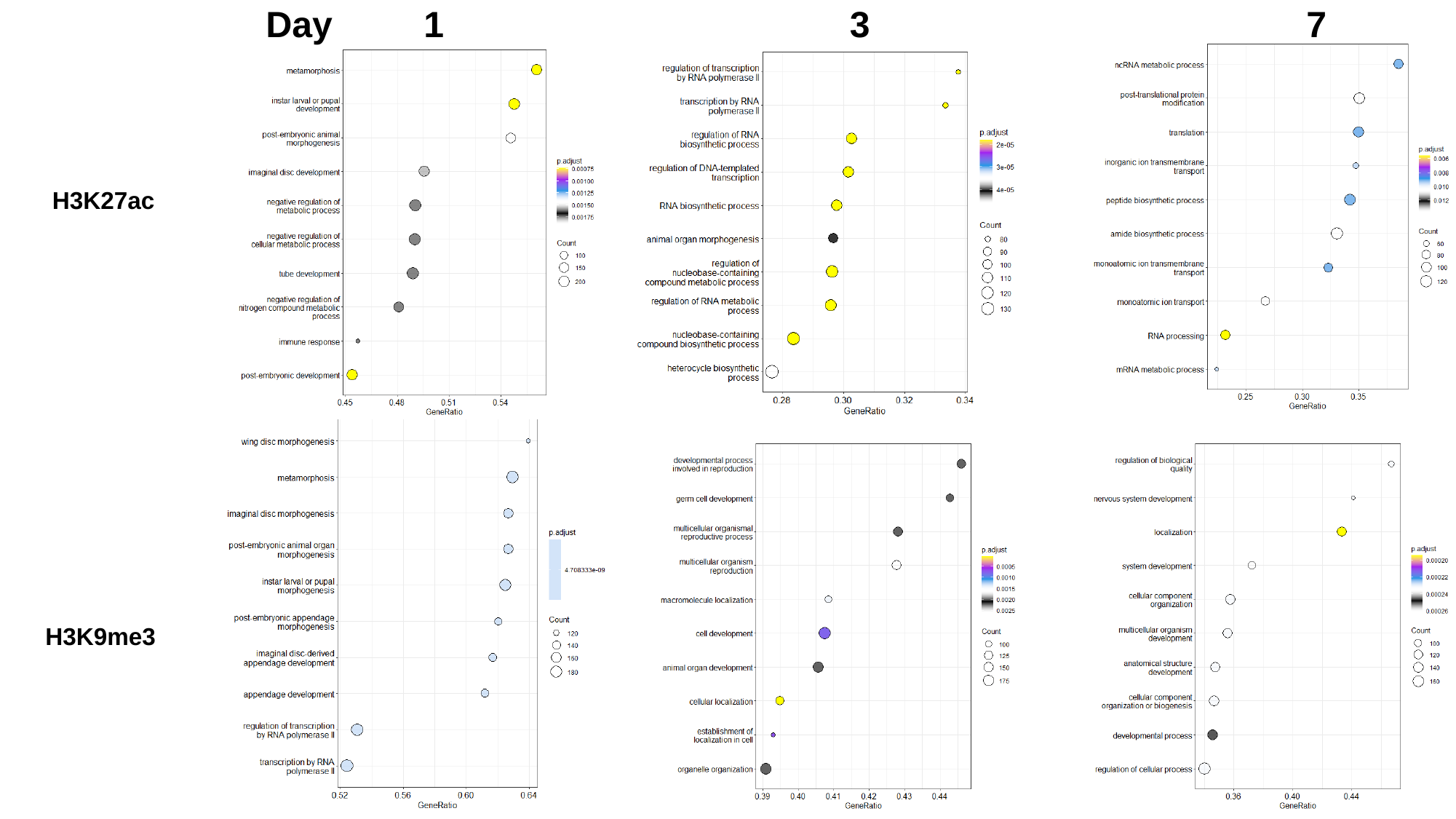

Day 1 3 7
 H3K27ac
 H3K9me3

## Slide 7
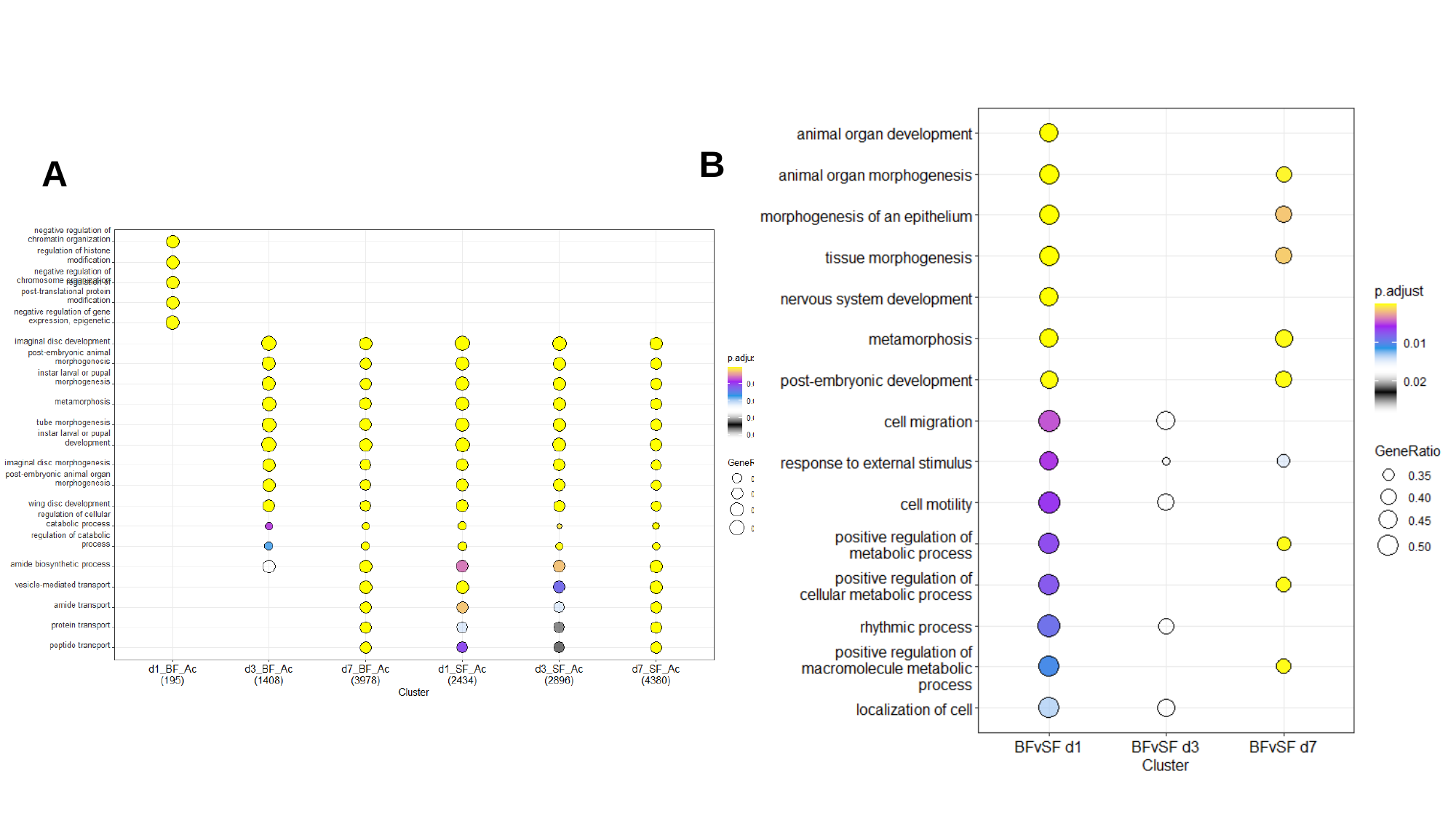

B
A

## Slide 8
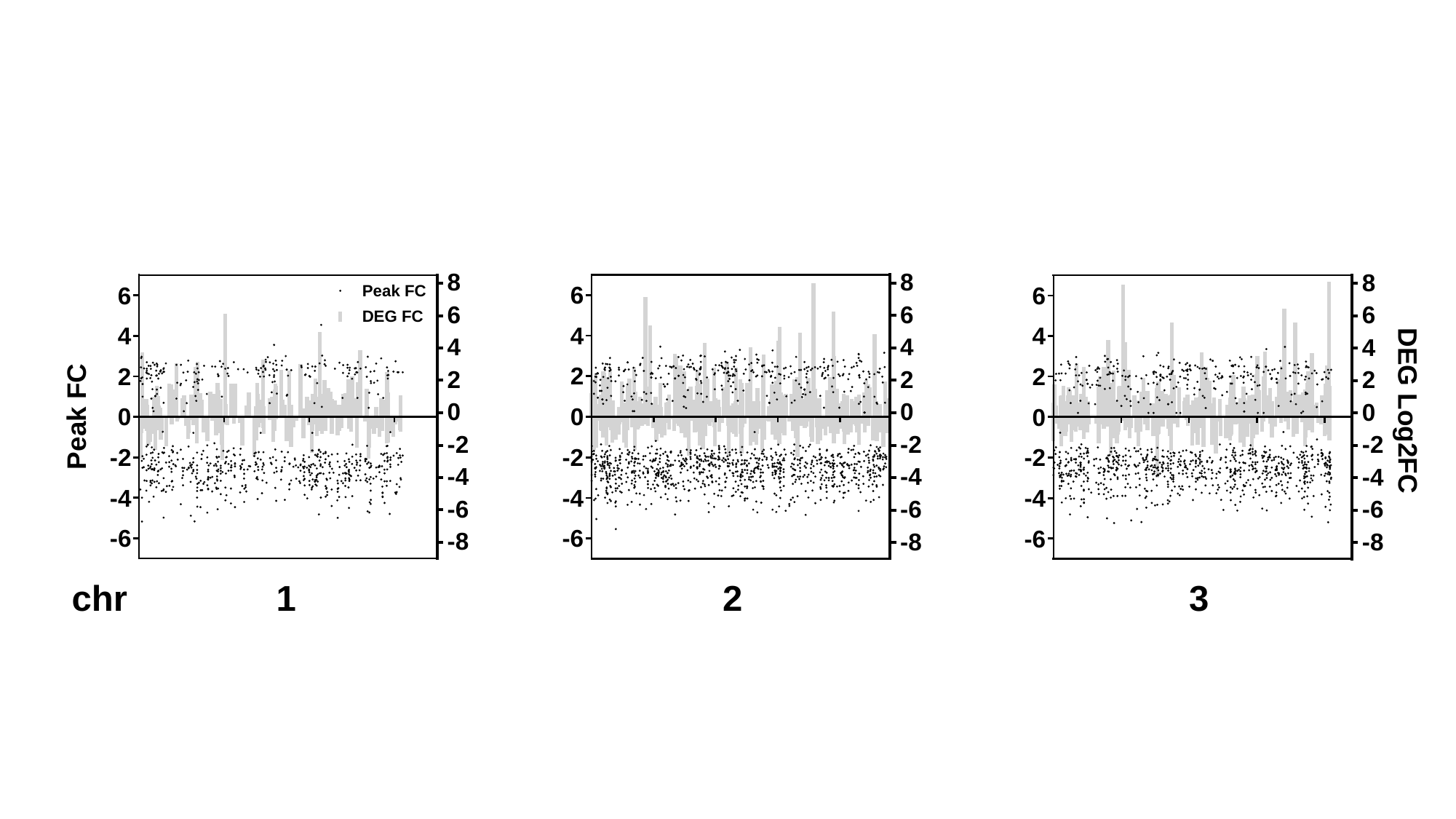

## Slide 9
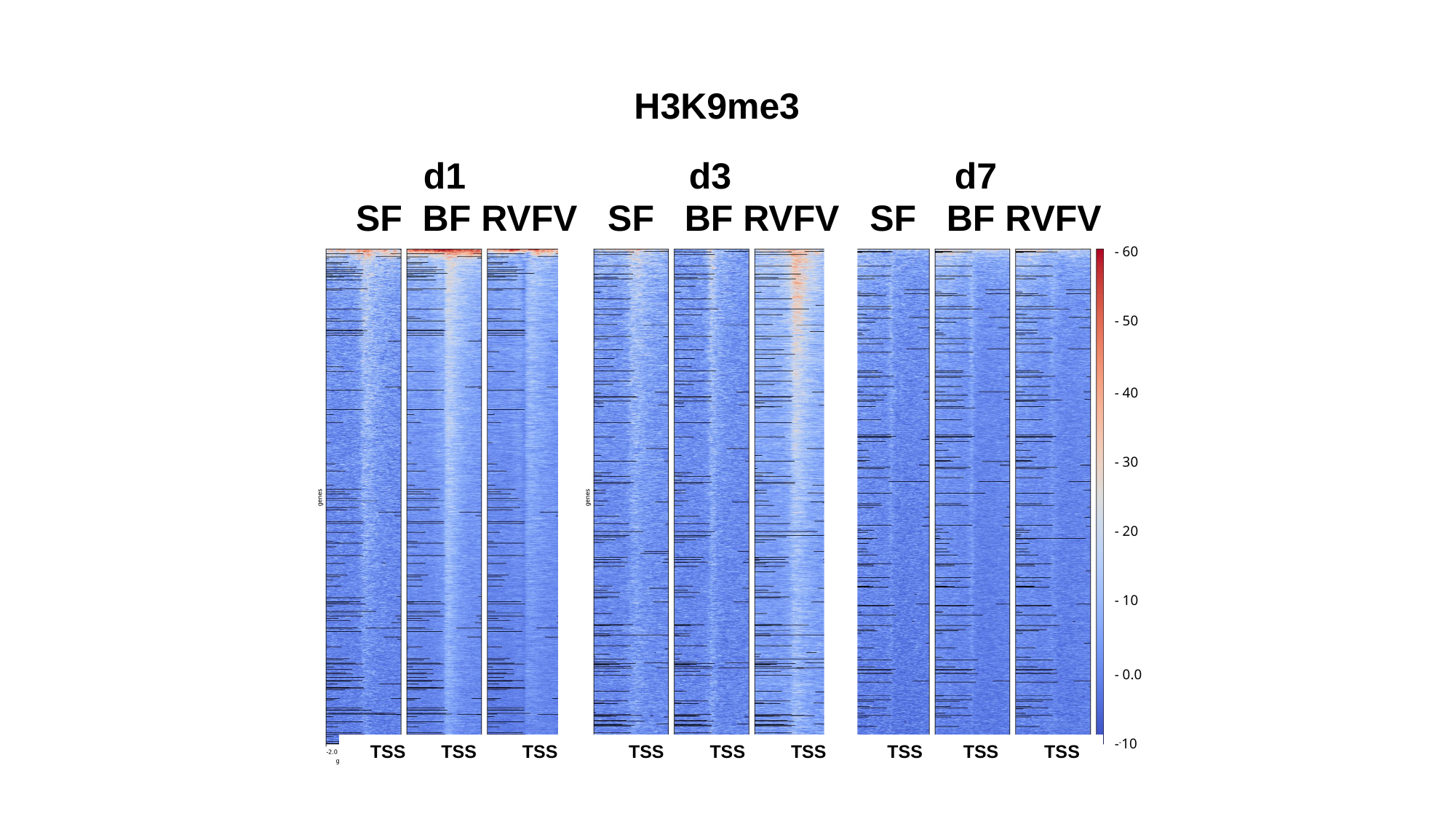

H3K9me3
H3K9me3
# d1 d3 d7
SF BF RVFV SF BF RVFV SF BF RVFV
- 60
- 50
- 40
- 30
- 20
- 10
- 0.0
--10
 TSS TSS TSS TSS TSS TSS TSS TSS TSS

## Slide 10
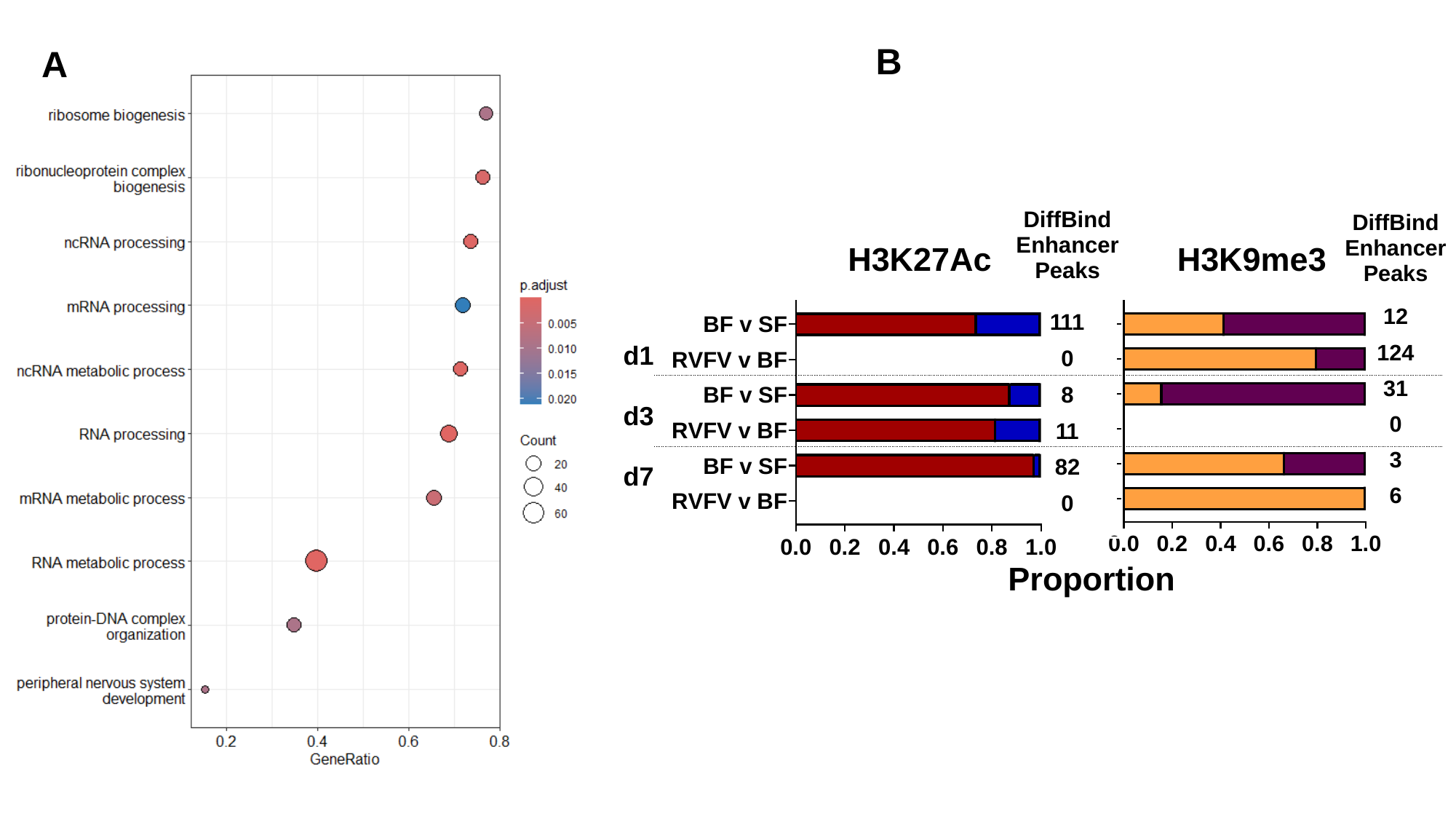

B
A
